# Mediating effect of coping style between family resilience and demoralization in patients with decompensated cirrhosis

**DOI:** 10.1038/s41598-026-49595-9

**Published:** 2026-04-21

**Authors:** Haixia Gao, Gang Mao, Shanshan Yin, Jianhua Niu, Ran Ding, Lei Liu

**Affiliations:** 1 Department of Nursing, Shandong First Medical University (Shandong Academy of Medical Sciences), Tai’an, 271016 Shandong China; 2 Department of Critical Care Medicine, The Fourth People’s Hospital of Jinan, Jinan, 250031 Shandong China; 3 Organization and Personnel Section, The Fourth People’s Hospital of Jinan, Jinan, 250031 Shandong China; 4 Department of Hematology, The Fourth People’s Hospital of Jinan, Jinan, 250031 Shandong China; 5 Department of Gastroenterology, The Fourth People’s Hospital of Jinan, Jinan, 250031 Shandong China

**Keywords:** Liver cirrhosis, Decompensated, Demoralization, Family resilience, Coping style, Mediation analysis, Diseases, Gastroenterology, Health care, Risk factors

## Abstract

This study aimed to examine the relationship between family resilience and demoralization syndrome (DS) in patients with decompensated cirrhosis and explore the mediating role of coping styles in this association. A survey of 260 patients with decompensated cirrhosis was conducted in the Department of Gastroenterology of a 3 A hospital in Jinan from September 2024 to March 2025 using the Mandarin Version of the Demoralization Scale (DS-MV), Chinese version of the Family Resilience Assessment Scale (FRAS-C) and Medical Coping Style Scale (MCMQ). Descriptive analysis and nonparametric test were performed using Free Statistics software version 2.2. Spearman correlation analysis and regression analysis were conducted with SPSS 27.0. The structural equation model was constructed via AMOS 28.0. The median DS score in patients with liver cirrhosis was 22, with an interquartile range (IQR) of 7–28. The proportion of patients with severe DS was 18.46%. Spearman correlation analysis demonstrated that DS was negatively correlated with family resilience, confrontation, and avoidance and positively correlated with acceptance-resignation (*r* = -0.668, *p* < 0.01; *r* = -0.314, *p* < 0.01; *r* = -0.162, *p* < 0.01; *r* = 0.540, *p* < 0.01). Mediation analysis revealed that acceptance-resignation partially mediated the relationship between family resilience and DS, accounting for 35.4% of the total effect (*p* < 0.001). Acceptance-resignation was identified as a partial mediator of the relationship between family resilience and DS. Therefore, attention should be paid to DS in patients with decompensated cirrhosis. Family resilience can also be used to reduce DS levels.

## Introduction

Liver cirrhosis is prevalent worldwide, and decompensated cirrhosis is a progressive, life-threatening, severe liver disease with a high mortality rate^1–3^. Its associated complications, such as ascites, gastrointestinal bleeding, and hepatic encephalopathy^4,5^, impose significant medical expenses and economic burdens on patients and difficulties in social participation caused by disease stigma all significantly reduce their quality of life^6,7^. These factors render patients with decompensated cirrhosis highly susceptible to negative emotions during the course of their illness^8^, which may lead to the development of demoralization syndrome (DS).

DS is characterized by a persistent inability to cope, helplessness, hopelessness, a sense of existential meaninglessness, and perceived incompetence^9,10^. DS is prevalent in patients with progressive disease or cancer^11^, and is associated with sleep disturbances, reduced quality of life, and suicidal ideation^12^. Current evidence suggests that liver cirrhosis can induce severe psychological distress and depression, thereby increasing the risk of suicide^13^, which is closely related to the development of DS. Current DS research has primarily focused on patients with cancer and end-stage patients with other chronic diseases^11^. However, its mechanism of action in decompensated cirrhosis remains underexplored.

Family resilience refers to families’ ability to adapt positively by flexibly using both internal and external resources when confronted with adverse or traumatic experiences^14^. Family resilience is a positive variable that helps families cope with disease effectively and contributes to fewer negative emotions^15,16^. One study discovered a negative correlation between family resilience and DS in patients with cleft lip and palate. Family resilience can effectively mobilize both internal and external family resources, which helps patients adapt better to the disease and reduces the severity of demoralization^17^.

Medical coping style is a cognitive behavior that can be divided into three types: confrontation, avoidance, and acceptance-resignation. Confrontation is a positive coping style, while avoidance and acceptance-resignation are negative coping styles^18^. Medical coping styles can influence patients’ psychological health, and negative coping strategies can lead to negative emotions^19^. The avoidance coping style is a significant risk factor for psychological distress in patients^20^.

It has been reported that family resilience is positively correlated with positive coping style^21^. Strong family resilience enables families to seek external resources and collaboratively solve problems when confronted with stress, leading them to believe that their family can provide practical help to the patient. This may reduce the patients’ sense of helplessness and encourage them to face the disease positively.

A study identified the presence of demoralization syndrome in patients with decompensated cirrhosis and revealed its association with certain protective factors, including internal and external resources such as psychological resilience and social support^22^. Coping style is regarded as a key mediator that transforms environmental resources into psychological outcomes. However, the relationships among family resilience, coping styles, and demoralization syndrome in patients with decompensated cirrhosis have not yet been investigated.

Stress-coping theory proposes that when an organism confronts stressors, it initiates cognitive appraisal and coping mechanisms, where coping resources and coping capacity are correlated with cognitive appraisal^23^. Patients with high family resilience possess more coping resources, which in turn influences their selection of coping styles. When the intensity of stress exceeds coping resources and capacity, it will impair their physical and mental health. Therefore, based on the Stress-coping theory, we propose the following hypothesis:

### Hypothesis

Medical coping styles mediate the relationship between family resilience and DS.

## Materials and methods

### Study design and setting

A descriptive cross-sectional path analysis study was conducted from September 2024 to March 2025 in a Grade A tertiary comprehensive hospital in Jinan, Shandong Province, China.

### Study participants, sample size, and sampling

Patients with decompensated liver cirrhosis who met the study’s inclusion criteria and agreed to participate were provided with a questionnaire. The inclusion criteria for the study were (a) pathological confirmation of decompensated cirrhosis, (b) age ≥ 18 years, (c) clear consciousness, and (d) signed informed consent. The exclusion criteria included (a) histopathologically confirmed primary hepatocellular carcinoma, (b) clinical instability or rapid deterioration, (c) diagnosis of a mental disorder or communication barrier, and (d) experienced major life events in the past 3 months.

The sample size was calculated using PASS (v15.0). Given the use of multiple regression analysis, PASS’s corresponding effect size method yielded a minimum requirement of 236 participants, based on an effect size of 0.15, a power of 90%, and a statistical significance level of 5%. While no consensus exists regarding Structural Equation Modeling sample sizes, the literature suggests that 200–500 is generally appropriate^24^.

The participants were selected using convenience sampling. The researcher first explained the purpose of the study to patients with decompensated cirrhosis, obtained informed consent, and then distributed self-report questionnaires. During the process of completing the questionnaires, if the patients encountered any problems or had questions, the researcher provided the required guidance.

### Measures

#### Demographic and clinical characteristics

The demographic and clinical characteristics questionnaire included questions on sex, age, education, employment status, children, religion, marital status, cohabitation status, residence, monthly household income per capita, self-care ability, disease awareness, and time since diagnosis.

#### Demoralization syndrome

The Demoralization Scale-Mandarin version (DS-MV), translated from the demoralization scale developed by Kissane^25^, was used to measure demoralization levels in patients. The DS-MV consists of 24 items across five subscales: loss of meaning, helplessness, disheartenment, dysphoria and sense of failure. Items are rated on a 5-point scale from 0 (“strongly disagree”) to 4 (“strongly agree”). Items 1, 6, 12, 17, and 19 were reverse scored. The total scores range from 0 to 96, with the same high demoralization cutoff (> 30) as the original scale. Within this research, the Cronbach’s alpha of the total scale was 0.96.

#### Family resilience

It was assessed using the shortened Chinese version of the Family Resilience Assessment Scale (FRAS-C). This 32-item self-report instrument, developed by Sixbey and translated into Chinese by Li et al.^26,27^, adopted a 4-point Likert scale (where 1 stands for strongly disagree, 2 for disagree, 3 for agree, and 4 for strongly agree). It comprises three subscales: Family Communication and Problem Solving (FCPS), Utilizing Social Resources (USR), and Maintaining a Positive Outlook (MPO). Overall scores span from 32 to 128, with higher values signifying stronger family resilience. In this study, the Cronbach’s alpha for the total scale was 0.99.

#### Medical coping style

The Medical Coping Modes Questionnaire (MCMQ) assesses three dimensions of coping style: Confrontation, Avoidance, and Acceptance-Resignation^28^. This 20-item instrument uses a 4-point Likert scale with dimension scores calculated by summing the corresponding items. Score ranges are as follows: Confrontation (1–32), Avoidance (1–28), and Acceptance-Resignation (1–20), where higher scores indicate stronger preference for each coping style.

### Data analysis

Normality and homogeneity of variance tests were conducted. Data following a normal distribution are reported as mean ± standard deviation (SD), whereas skewed data are represented as interquartile range (IQR). Statistical analyses were performed using Free Statistics software version 2.2 for descriptive statistics and nonparametric test, and SPSS 27.0 for Spearman’s correlation analysis and multivariate linear regression analysis. IBM SPSS AMOS 28.0 was employed to construct the structural equation model. The maximum likelihood method was used to fit the models. Normality testing revealed that the data in this study exhibited a non-normal distribution, and the Bootstrap method was therefore used to adjust the data^29^.

### Ethical considerations

This study was approved by the Ethics Committee of the Fourth People’s Hospital of Jinan (Approval No. LL20240054). All methods were performed in accordance with the relevant guidelines and regulations, including the Declaration of Helsinki. Prior to the start of the investigation, patients with decompensated cirrhosis were informed of the study’s purpose and significance. The principles of voluntary study participation and data confidentiality were clarified for all participants. Additionally, obtaining written informed consent from each participant was mandatory.

## Results

### Sociodemographic characteristics of patients with decompensated liver cirrhosis

A total of 260 participants participated in this study. The majority were male (61.2%), and the largest proportion was in the 60–74-year age group (43.1%). Regarding educational attainment, most participants had completed junior high school or below (70.7%). Nearly half of the participants were farmers (49.6%). The vast majority reported having children (96.9%), with 1–2 children being the most common (82.7%). Nonparametric test results revealed that when comparing the demoralization syndrome scores of decompensated cirrhosis patients with different levels of self-care ability, there was a statistically significant difference (*p* < 0.05). The general demographic data of the patients with decompensated liver cirrhosis are shown in Table 1.

**Table 1:** Comparison of general conditions of patients with decompensated liver cirrhosis.

Variables	Total (*n* = 260)	0 (*n* = 212)	1 (*n* = 48)	*p*	Statistic
Gender, n (%)				0.440	0.596
Male	159 (61.2)	132 (62.3)	27 (56.2)		
Female	101 (38.8)	80 (37.7)	21 (43.8)		
Age, n (%)				0.826	Fisher
18–44	19 (7.3)	15 (7.1)	4 (8.3)		
45–59	108 (41.5)	86 (40.6)	22 (45.8)		
60–74	112 (43.1)	94 (44.3)	18 (37.5)		
≥ 75	21 (8.1)	17 (8.0)	4 (8.3)		
Education, n (%)				0.683	Fisher
Elementary school and below	89 (34.2)	75 (35.4)	14 (29.2)		
Junior high school	95 (36.5)	75 (35.4)	20 (41.7)		
Senior high school/vocational school	50 (19.2)	42 (19.8)	8 (16.7)		
Associate degree and above	26 (10.0)	20 (9.4)	6 (12.5)		
Employment status, n (%)				0.231	Fisher
Employed	43 (16.5)	36 (17.0)	7 (14.6)		
Retired	64 (24.6)	49 (23.1)	15 (31.2)		
Not in the workforce	24 (9.2)	17 (8.0)	7 (14.6)		
Farmer	129 (49.6)	110 (51.9)	19 (39.6)		
Children, n (%)				0.183	Fisher
0	8 (3.1)	6 (2.8)	2 (4.2)		
1	104 (40.0)	80 (37.7)	24 (50.0)		
2	111 (42.7)	92 (43.4)	19 (39.6)		
≥ 3	37 (14.2)	34 (16.0)	3 (6.2)		
Religion, n (%)				0.345	0.891
No	232 (89.2)	191 (90.1)	41 (85.4)		
Yes	28 (10.8)	21 (9.9)	7 (14.6)		
Marital status, n (%)				0.155	Fisher
Married or cohabiting	237 (91.2)	196 (92.5)	41 (85.4)		
Single/divorced/widowed	23 (8.8)	16 (7.5)	7 (14.6)		
Cohabitation status, n (%)				0.243	Fisher
Living alone	12 (4.6)	8 (3.8)	4 (8.3)		
Living with others	248 (95.4)	204 (96.2)	44 (91.7)		
Residence, n (%)				0.414	0.667
Rural	127 (48.8)	101 (47.6)	26 (54.2)		
City	133 (51.2)	111 (52.4)	22 (45.8)		
Monthly household income per capita, n (%)				0.786	1.063
<1500	64 (24.6)	50 (23.6)	14 (29.2)		
1500–1999	35 (13.5)	28 (13.2)	7 (14.6)		
2000–2999	54 (20.8)	46 (21.7)	8 (16.7)		
≥ 3000	107 (41.2)	88 (41.5)	19 (39.6)		
Disease awareness, n (%)				0.268	1.226
Fully informed	195 (75.0)	162 (76.4)	33 (68.8)		
Partially informed	65 (25.0)	50 (23.6)	15 (31.2)		
Self-care ability, n (%)				0.009	Fisher
No dependency	223 (85.8)	187 (88.2)	36 (75.0)		
Mild dependency	23 (8.8)	18 (8.5)	5 (10.4)		
Moderate dependency	13 (5.0)	6 (2.8)	7 (14.6)		
Severe dependency	1 (0.4)	1 (0.5)	0 (0)		
Time since diagnosis, n (%)				0.209	Fisher
< 0.5 years	30 (11.5)	26 (12.3)	4 (8.3)		
0.5–1 years	24 (9.2)	21 (9.9)	3 (6.2)		
1 –5 years	81 (31.2)	58 (27.4)	23 (47.9)		
6– 10 years	46 (17.7)	39 (18.4)	7 (14.6)		
11– 20 years	49 (18.8)	43 (20.3)	6 (12.5)		
> 20 years	30 (11.5)	25 (11.8)	5 (10.4)		

1: Total score of DS > 30; 0: Total score of DS ≤ 30.

### Scores of DS, family resilience, and coping style of patients with decompensated liver cirrhosis

The median total DS score was 22, with an interquartile range (IQR) of 7 to 28, and sense of failure had the highest median score of 6 points (IQR, 5–7). The median total score for family resilience was 97, with an interquartile range (IQR) of 95 to 124. For the coping style subscales, the median scores (IQR) were as follows: confrontation, 24 (21–26); avoidance, 17 (16–18); and acceptance-resignation, 5 (5–6.26). The DS, family resilience, and coping style scores of decompensated cirrhotic patients are shown in Table 2.

**Table 2:** Scores of demoralization syndrome, family resilience and coping style of patients with decompensated liver cirrhosis.

Variable	M(P25 ~ P75)	Item count
Demoralization syndrome	22(7–28)	24
Disheartenment	5(1–7)	6
Loss of meaning	3(0–5)	5
Dysphoria	4(0–6)	5
Helplessness	3(0–4)	4
Sense of failure	6(5–7)	4
Family resilience	97(95–124)	32
FCPS	70(68–90)	23
USR	9(9–11)	3
MPO	18(18–24)	6
Coping style	–	20
Confrontation	24(21–26)	8
Avoidance	17(16–18)	7
Acceptance-resignation	5(5–6.26)	5

### Correlation analysis of DS, family resilience, and coping style of patients with decompensated liver cirrhosis

Family resilience of patients with decompensated liver cirrhosis was significantly negatively correlated with demoralization syndrome (*r* = -0.668, *p* < 0.01), and with acceptance-resignation (*r* = -0.450, *p* < 0.01). Acceptance-resignation was significantly positively correlated with DS (*r* = 0.540, *p* < 0.01). Confrontation and avoidance were significantly negatively correlated with DS (*r* = -0.314, *p* < 0.01; *r* = -0.162, *p* < 0.01). The correlations among DS, family resilience, and coping styles are shown in Table 3.

**Table 3:** Correlation analysis of demoralization syndrome, family resilience and coping style of patients with decompensated liver cirrhosis.

Correlations	1	2	3	4	5	6	7	8	9	10	11	12	13
DS	1												
Loss of meaning	0.884**	1											
Dysphoria	0.898**	0.768**	1										
Helplessness	0.916**	0.868**	0.851**	1									
Sense of failure	0.809**	0.632**	0.611**	0.663**	1								
Disheartenment	0.928**	0.800**	0.816**	0.819**	0.695**	1							
Family resilience	− 0.668**	− 0.610**	− 0.600**	− 0.624**	− 0.573**	− 0.644**	1						
FCPS	− 0.660**	− 0.610**	− 0.598**	− 0.619**	− 0.558**	− 0.639**	0.989**	1					
USR	− 0.548**	− 0.488**	− 0.468**	− 0.503**	− 0.506**	− 0.530**	0.805**	0.756**	1				
MPO	− 0.671**	− 0.607**	− 0.616**	− 0.619**	− 0.565**	− 0.654**	0.928**	0.914**	0.733**	1			
Confrontation	− 0.314**	− 0.255**	− 0.199**	− 0.251**	− 0.419**	− 0.272**	0.347**	0.332**	0.443**	0.290**	1		
Avoidance	− 0.162**	− 0.144*	− 0.183**	− 0.164**	− 0.142*	− 0.112	0.141*	0.130*	0.171**	0.104	0.140*	1	
Acceptance-resignation	0.540**	0.478**	0.423**	0.484**	0.514**	0.551**	− 0.450**	− 0.439**	− 0.431**	− 0.438**	− 0.506**	− 0.108	1

**p < 0.01, *p < 0.05.

### Regression analysis

All variance inflation factors (VIF) were below 5, indicating no multicollinearity among the independent variables. Multiple linear regression analysis revealed that family resilience, avoidance, and acceptance-resignation were significant predictors of demoralization syndrome in patients with decompensated liver cirrhosis (*p* < 0.05). The model explained 67.5% of the variance in DS among the patients with decompensated cirrhosis. The results of the multiple linear regression analysis are presented in Table 4.

**Table 4:** Results of multiple linear regression analysis.

Variable	B	SE	β	t	*p*	Tolerance	VIF	sr
Constant	53.623	7.039		7.618	<0.001			
Self-care ability	0.882	1.018	0.032	0.867	0.387	0.935	1.070	0.031
Family resilience	− 0.395	0.035	− 0.484	− 11.296	<0.001	0.683	1.465	− 0.400
Confrontation	0.128	0.160	0.035	0.799	0.425	0.641	1.560	0.028
Avoidance	− 0.634	0.255	− 0.090	− 2.487	0.014	0.967	1.034	− 0.088
Acceptance-resignation	2.316	0.247	0.460	9.389	<0.001	0.523	1.913	0.333

R^2^ = 0.681, adjusted R^2^ = 0.675, F = 108.489, VIF < 5, no significant covariance in the independent variables and normally distributed residuals.

### Testing the hypothesized model

**Fig. 1:**
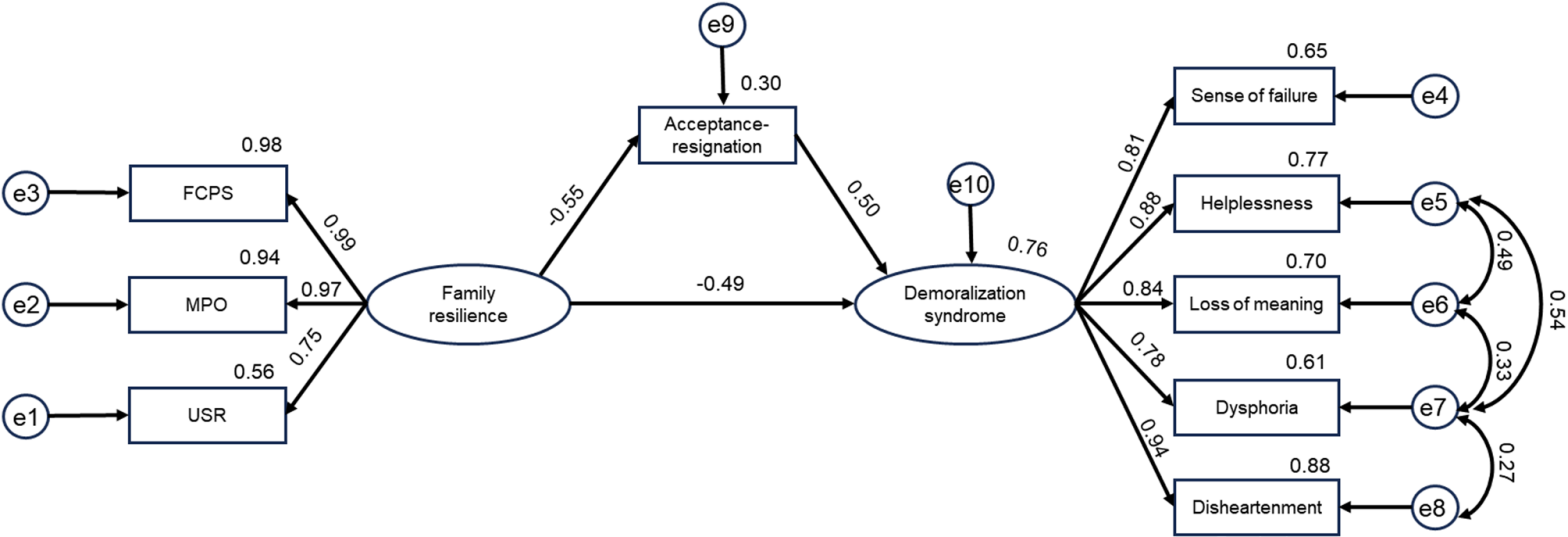
The mediating effect of acceptance-resignation in the relationship between family resilience and demoralization syndrome in decompensated cirrhotic patients (Standard coefficients).

The structural equation model incorporating confrontation did not achieve acceptable model fit after modification. In the model incorporating avoidance, neither the path from family resilience to avoidance nor that from avoidance to demoralization syndrome reached statistical significance (*p* > 0.05). The structural equation model incorporating acceptance-resignation was ultimately constructed and retained. The model fit results are shown, *χ*^*2*^*/ df* = 2.510, NFI = 0.980, IFI = 0.988, GFI = 0.957, RFI = 0.965, TLI = 0.979, CFI = 0.987, RMSEA = 0.076. Table 5 shows that the three path coefficients are statistically significant (*p* < 0.001). Family resilience was directly negatively associated with DS (β = -0.494, *p* < 0.001) and negatively associated with acceptance-resignation (β = -0.546, *p* < 0.001). Acceptance-resignation was significantly positively associated with DS (β = 0.496, *p* < 0.001). The results indicated that acceptance-resignation had a significant mediating effect on the relationship between family resilience and DS in patients with decompensated liver cirrhosis. The results are shown in Fig. 1. The hypothesis was further verified by revealing the mediating effect of acceptance-resignation on the relationship between family resilience and demoralization syndrome.

**Table 5:** Path analysis of structural equation model.

Path	Standardized Coefficients	Unstandardized Coefficients	SE	CR	*p*
Family resilience→ Acceptance-resignation	− 0.546	− 0.471	0.046	− 10.243	***
Acceptance-resignation→ Demoralization syndrome	0.496	0.416	0.041	10.127	***
Family resilience→ Demoralization syndrome	− 0.494	− 0.358	0.035	− 10.164	***

***p < 0.001.

### Direct and indirect effects

Table 6 summarizes the direct, indirect, and total estimates of the model paths. Family resilience had a significant direct effect (B = -0.358, *p* < 0.001), accounting for 64.6% of the total effect. The indirect effects were mediated through acceptance-resignation (B = -0.196, *p* < 0.001). The indirect effect explained 35.4% of the total effect.

**Table 6:** Total, direct, and indirect effect.

Items	Coeff/effect	Proportion	*p*	95%CI	
				Lower	Upper
Direct effect	− 0.358	64.6%	< 0.001	− 0.436	− 0.289
Indirect effect	− 0.196	35.4%	< 0.001	− 0.277	− 0.123
Total effect	− 0.554	100%	< 0.001	− 0.653	− 0.452

## Discussion

The purpose of this study was to explore the relationship between family resilience and demoralization syndrome in patients with decompensated cirrhosis and to explore the mediating role of coping styles. The finding that family resilience was negatively associated with DS was consistent with previous studies^17^. This study also found that acceptance-resignation mediates the relationship between family resilience and DS. These findings are of great significance for improving negative emotions in patients with decompensated cirrhosis. It is necessary to take measures to enhance family resilience in this population and to modify patients’ coping styles.

In the present study, the median total demoralization syndrome score was 22, which was lower than the score in the study by Li, who found that the DS-MV score of inpatients with liver cirrhosis was (37.92 ± 12.85)^30^. Patients with decompensated cirrhosis gradually accept reality during the long-term recurrent course of the disease, which alleviates their fear and worry about the disease. The questionnaire survey was conducted when the disease was stable, at which point the disease had the least impact on the patients’ psychological state. In addition, patients may have concealed their negative emotions when completing a questionnaire.

The median total score for family resilience was 97, with an interquartile range (IQR) of 95 to 124, higher than that reported in a previous study^31^. Studies have shown that strong resilience enables families and individuals to effectively adapt to illness^32^, fostering stability in family functioning, and facilitating patient recovery. The low degree of demoralization in patients with decompensated cirrhosis observed in this study may be related to this finding. Therefore, healthcare professionals are advised to reduce DS by enhancing family resilience. Some research teams have conducted studies aimed at improving family resilience. For instance, Yang et al. implemented interventions targeted at the family members of patients with gynecologic malignancies, significantly enhancing patients’ family resilience^33^. FOCUS is a concise family intervention program that provides families with a platform for discussion. Studies indicate that families participating in this program reported improved family resilience^34^.

The results showed that 209 participants (80.4%) adopted confrontation, 33 (12.7%) adopted avoidance, and 18 (6.9%) adopted acceptance-resignation. Adaptive coping style facilitates patients’ active collaboration during treatment and prompt reporting of changes in their condition, thereby contributing to improved disease control. Avoidance and acceptance-resignation coping styles may be associated with recurrent disease episodes, suboptimal treatment outcomes, and a significant financial burden. Correlation analysis in this study revealed a negative correlation between avoidance and demoralization syndrome, while regression analysis showed that avoidance negatively predicted demoralization syndrome. According to the stress-coping theory, when an organism is exposed to stress, a cascade of cognitive appraisal processes and coping mechanisms is activated. Cognitive appraisal is closely associated with an individual’s coping capacity and available coping resources^23^. Decompensated liver cirrhosis is characterized by chronic progressive deterioration and refractory symptoms. In such uncontrollable clinical circumstances, avoidance may serve an adaptive, short-term protective role. Health education equips patients with disease-specific knowledge, enhances their self-management capabilities, and serves as a foundation for adaptive coping^35^. Therefore, healthcare providers should offer patients more disease-related health education and psychological counseling and encourage them to maintain a proactive coping state.

The results showed that family resilience and acceptance-resignation were predictors of demoralization syndrome. Family resilience enables families to utilize social resources better and maintain positive attitudes toward coping with stress. Studies have found that favorable family resilience can also enhance patients’ psychological resilience, enabling them to face the disease optimistically, thus reducing the severity of DS^22^. A direct negative association between family resilience and DS was identified, and an indirect association between them via acceptance-resignation was also found in the structural equation model. This study found that family resilience was positively correlated with positive coping style, which is consistent with the conclusions drawn by Zhang^36^. Lower family resilience may provide patients with less support, making them feel helpless and consequently abandon resistance, adopting an acceptance-resignation coping style which further exacerbates demoralization. However, causal relationships cannot be inferred from a cross-sectional design, and future longitudinal studies could be conducted to explore the causal relationships between the variables.

This survey on family resilience among patients with decompensated cirrhosis was conducted in the context of Chinese culture and may be influenced by Chinese family cultural values. In the context of China’s family-centered culture, the family serves as a spiritual anchor for its members and functions as a community of shared resources and mutual responsibility^37,38^. Within such families, family resilience can be enhanced through intergenerational support and shared economic burdens, reflecting a cultural orientation distinct from Western contexts. Support and care among family members also influence how patients cope with illness. Strong family resilience enhances the overall capacity of both patients and their families to cope with the disease. It facilitates a collective response to illness-related challenges, helps patients cultivate a positive attitude and psychological resilience in adapting to their condition, and thereby mitigates the negative emotions. In terms of coping styles, patients not only consider how to manage their illness individually but also take into account the family’s financial capacity to afford treatment. On some occasions, patients may prioritize the family’s overall interests over continuing their own treatment, which should not be simplistically categorized as passive coping. This family-centered approach stands in marked contrast to the individualistic orientation prevalent in Western cultures, where family resilience emphasizes personal independence and independent coping capacity, and patients tend to make medical decisions autonomously. Future studies could explore the relationship between family resilience and DS in the context of Western culture.

This study recruited participants using a convenience sampling method, which may introduce selection bias. Specifically, patients with higher levels of demoralization syndrome or those adopting acceptance-resignation coping style may be unwilling or unable to participate in the study due to psychological distress and reduced motivation. This bias may lead to an underestimation of the strength of the associations between family resilience, coping styles and demoralization syndrome, making the observed effect size lower than the true level. Regarding the direction of correlation, this bias does not change the direction of the correlation between variables.

Based on the relationships between family resilience, coping styles, and demoralization syndrome identified in this study, we propose an intervention framework to reduce demoralization in patients with decompensated cirrhosis. First, healthcare professionals should monitor patients’ psychological status and, when necessary, assess those with low mood for demoralization syndrome. Interventions such as dignity therapy and logotherapy should be provided for patients with severe symptoms^39,40^. Second, family-focused intervention programs should be implemented to enhance patients’ family resilience^33,34^. Finally, healthcare providers should guide patients in facing their illness appropriately, offer moderate psychological support, and help them adopt more positive coping style.

In summary, this study revealed a link between family resilience, the acceptance-resignation coping style, and DS. Enhancing family resilience and adopting proactive coping style toward the disease are crucial for maintaining a positive mindset in patients with decompensated cirrhosis.

### Limitations

Several limitations exist in this study. Firstly, self-reported questionnaires were used as the data collection tool, which might introduce reporting bias. Secondly, causal relationships cannot be inferred from a cross-sectional design. Future longitudinal studies could be conducted to verify potential causal relationships. Finally, this study adopted convenience sampling for participant recruitment. Patients with higher levels of demoralization or those adopting acceptance-resignation coping style might be unwilling or unable to participate in the study due to hopelessness or reduced motivation, which may lead to an underestimation of the relationship between family resilience, coping styles, and demoralization. Future studies should adopt more representative sampling methods to verify the current findings.

## Conclusion

This study found a direct negative correlation between family resilience and demoralization syndrome in patients with decompensated cirrhosis, and the acceptance-resignation coping style mediated the effect of family resilience on DS to a certain extent. These findings provide a theoretical foundation for targeted interventions aimed at reducing demoralization in patients with decompensated cirrhosis. Effective strategies should integrate family resilience with coping styles.

## Data Availability

The data that support the findings of this study are available from the corresponding author upon reasonable request.
